# Closing Editorial: Editorial Board Members’ Collection Series: Optical Measurements and Sensing Technology

**DOI:** 10.3390/s26196040

**Published:** 2026-09-23

**Authors:** Michele Norgia, Elfed Lewis, Alberto Vallan

**Affiliations:** 1 Dipartimento di Elettronica, Informazione e Bioingegneria, Politecnico di Milano, Via Ponzio 34/5, 20133 Milano, Italy; 2 Optical Fibre Sensors Research Centre (OFSRC), University of Limerick, V94 T9PX Limerick, Ireland; elfed.lewis@ul.ie; 3 Department of Electronics and Telecommunications, Politecnico di Torino, Corso Duca degli Abruzzi, 24, 10129 Torino, Italy; alberto.vallan@polito.it

## 1. Introduction

This editorial provides closing remarks for the Special Issue on “Optical Measurements and Sensing Technology” published in *Sensors*. This Special Issue was established to offer a comprehensive venue for sharing state-of-the-art research in this fast-developing field. Optical sensors are now widespread in countless scientific and industrial applications, offering optimal metrological performance through non-contact measurement techniques. [1]. The discipline of optical sensing includes numerous approaches, from traditional interferometry [2,3,4,5] to custom sensors for specific applications [6,7,8] or state-of-the-art physics sensors, such as quantum-enhanced methods [9,10] and frequency combs [11], all contributing to improved accuracy in the measurement of physical quantities [12]. As we move further into the era of the second quantum revolution, optical sensing systems are leading technological progress, supporting innovations in areas spanning fundamental physics to advanced manufacturing [13].

We are pleased to report on the successful completion of this Special Issue, which has brought together eight high-quality research contributions covering fiber-optic sensors, biomedical applications, vision in foggy conditions, an optical E-field sensor, Optical Time-Domain Reflectometry (OTDR) and spectrometry. These articles confirm the continuous rapid development of optical sensing techniques and their growing importance in measurement applications.

## 2. An Overview of Published Articles

The contributions in this Special Issue cover various topics concerning optical sensors and their applications. Collectively, these contributions tackle key challenges and opportunities in optical sensing, covering both fundamental studies and real-world applications. In just a few months, they have attracted more than 20,000 views and over 35 citations, highlighting their strong impact and relevance within the wider scientific community.

The article by Tapetado and Vázquez [LC1] presents a new model for describing the behavior of a fiber-optic pyrometer in the presence of a tilting angle with respect to the normal of the emitted surface. The results of the work allow the prediction and compensation of potential temperature measurement errors for this kind of sensor.

The second text published in this Special Issue [LC2] focuses on the experimental characterization of the polarization behavior of light backscattered under foggy conditions. The obtained results are useful for optimizing optical sensors working with active polarimetric imaging techniques for outdoor applications.

The research from Fasani et al. [LC3] delves into the application of an optical E-field sensor to diagnose the internal defects of a set of composite insulators. The results show that the sensor can successfully detect the presence of an internal defect, since its presence distorts the E-field profile when compared to the profile of a sound insulator.

The fourth text published in this Special Issue [LC4] describes a fiber-optic ball resonator able to detect a cancer biomarker in situ: the CD44 protein. The realized sensor is insensitive to pressure changes and is highly selective to the targeted protein, hence improving patient diagnosis and monitoring of malignant tumors.

The article by Gritsenko et al. [LC5] demonstrates a distributed acoustic sensor based on phase-sensitive OTDR for smart home applications. The distributed fiber-optic microphone signal was elaborated by taking advantage of an experimentally verified mathematical model to achieve optimum speech recognition quality.

In the sixth article [LC6], Anelli, Pellicorio, Bello and Merlo present a low-cost optical sensor for the detection of urea concentration. The proposed sensor is based on the absorption properties of urea in the near-infrared wavelength region: they demonstrated that the transmitted intensity at two wavelengths is linearly related to urea concentration in water over a wide range and with good sensitivity.

The study by McCluskey, van Klinken and Fiore focuses on new figure of merit, the integrated spectral sensitivity, for spectral transducers used in optical sensing. The figure of merit is directly related to the physical limit of precision given by the Cramér–Rao lower bound, and is applicable to a wide variety of sensing systems. The obtained results are beneficial for the general design of optical sensors.

The last contribution, by Li et al., reports a study on inorganic scintillator materials applied to optical fiber radiation sensing probes. A theoretical model for luminescence coupling into fibers, as a function of numerical aperture, is proposed and demonstrated experimentally. The results indicate how optical fiber radiation probes can be optimized; for example, it is demonstrated that inorganic scintillators for end-constructed probes do not need to be excessively long.

## 3. Impact and Future Directions

The various topics covered in this Special Issue demonstrate the extensive scope of the optical sensor technology field. The exchange of concepts and methodological approaches across different research areas strengthens the field and fosters faster technological progress. The considerable interest shown by readers, demonstrated by more than 20,000 views, highlights the widespread significance of optical sensing technologies and underscores the value of open access publication in promoting the dissemination of scientific knowledge.

Looking to the future, we expect the development of innovative measurement configurations, enhanced by increasingly advanced analysis and processing systems also exploiting the enormous potential of artificial intelligence applied to signal processing for optical sensors [14,15].

## 4. Conclusions

This Special Issue “Optical Measurements and Sensing Technology” provides an overview of different optical sensing techniques. The papers included in this Special Issue highlight the diversity and dynamic nature of optical sensing research, encompassing both fundamental theoretical developments and practical applications. Taken together, these contributions illustrate the ongoing progress of optical sensing technologies and emphasize their impact on scientific advancement, technological innovation, and industrial development.

We would like to extend our appreciation to all the authors for their outstanding contributions, to the reviewers for their careful evaluations and insightful comments, and to the editorial staff of *Sensors* for their dedicated support and professionalism throughout the development of this Special Issue. We believe that the studies collected in this issue will provide an important reference for researchers in the field and encourage further advances in optical sensing techniques.
